# Epidemiology of cholera outbreaks and socio-economic characteristics of the communities in the fishing villages of Uganda: 2011-2015

**DOI:** 10.1371/journal.pntd.0005407

**Published:** 2017-03-13

**Authors:** Godfrey Bwire, Aline Munier, Issaka Ouedraogo, Leonard Heyerdahl, Henry Komakech, Atek Kagirita, Richard Wood, Raymond Mhlanga, Berthe Njanpop-Lafourcade, Mugagga Malimbo, Issa Makumbi, Jennifer Wandawa, Bradford D. Gessner, Christopher Garimoi Orach, Martin A. Mengel

**Affiliations:** 1 Department of Community Health, Ministry of Health (MOH), Kampala, Uganda; 2 Agence de Médecine Préventive (AMP), Paris, France; 3 Agence de Médecine Préventive (AMP), Abidjan, Cote d’Ivoire; 4 Department of Community and Behavioral Sciences, Makerere University School of Public Health (MUSPH), Kampala, Uganda; 5 National Health Laboratory Services, Ministry of Health, Kampala, Uganda; 6 Agence de Médecine Préventive (AMP), Ferney-Voltaire, France; 7 National Disease Control Department, Ministry of Health, Kampala, Uganda; 8 Health Emergency Operation Centre (EOC), Ministry of Health, Kampala, Uganda; 9 Department of Health, Mbale District Local Government, Mbale, Uganda; Massachusetts General Hospital, UNITED STATES

## Abstract

**Background:**

The communities in fishing villages in the Great Lakes Region of Africa and particularly in Uganda experience recurrent cholera outbreaks that lead to considerable mortality and morbidity. We evaluated cholera epidemiology and population characteristics in the fishing villages of Uganda to better target prevention and control interventions of cholera and contribute to its elimination from those communities.

**Methodology/Principal findings:**

We conducted a prospective study between 2011–15 in fishing villages in Uganda. We collected, reviewed and documented epidemiological and socioeconomic data for 10 cholera outbreaks that occurred in fishing communities located along the African Great Lakes and River Nile in Uganda. These outbreaks caused 1,827 suspected cholera cases and 43 deaths, with a Case-Fatality Ratio (CFR) of 2.4%. Though the communities in the fishing villages make up only 5–10% of the Ugandan population, they bear the biggest burden of cholera contributing 58% and 55% of all reported cases and deaths in Uganda during the study period. The CFR was significantly higher among males than females (3.2% vs. 1.3%, p = 0.02). The outbreaks were seasonal with most cases occurring during the months of April-May. Male children under age of 5 years, and 5–9 years had increased risk. Cholera was endemic in some villages with well-defined “hotspots”. Practices predisposing communities to cholera outbreaks included: the use of contaminated lake water, poor sanitation and hygiene. Additional factors were: ignorance, illiteracy, and poverty.

**Conclusions/Significance:**

Cholera outbreaks were a major cause of morbidity and mortality among the fishing communities in Uganda. In addition to improvements in water, sanitation, and hygiene, oral cholera vaccines could play an important role in the prevention and control of these outbreaks, particularly when targeted to high-risk areas and populations. Promotion and facilitation of access to social services including education and reduction in poverty should contribute to cholera prevention, control and elimination in these communities.

## Author summary

Cholera, though a preventable and treatable disease, remains a major cause of morbidity and mortality in the Great Lakes Region of Africa, including Uganda. The communities in the fishing villages constitute 5–10% of the total Ugandan population. Most fishing villages are located along Lakes Victoria, Albert and Edward and the River Nile. During the study period, 2011–2015 these villages were responsible for over 50% of the reported annual cholera cases and deaths in Uganda. The CFR was significantly higher among males than females (3.2% vs. 1.3%, p = 0.02). Our study is the first to systematically describe the epidemiology of these outbreaks and socioeconomic characteristics of communities in the fishing villages in Uganda. Our study found that persons in the fishing villages were at increased risk of cholera outbreaks due to poor access to safe water, sanitation, and hygiene. Furthermore, the villages had similar population characteristics such as illiteracy, ignorance regarding cholera transmission, poverty and constant population migration. In addition to improvements in water, sanitation, and hygiene, complementary use of oral cholera vaccines could play an important role, particularly when targeted to high-risk areas and populations. As a long term strategy, improvements in education and reduction in poverty should contribute to cholera prevention, control and elimination in the fishing villages and Uganda as whole.

## Introduction

Cholera remains a major public health problem in many countries in sub-Saharan Africa. In 2014, 190,549 cases and 2,231 deaths were reported to the World Health Organization (WHO). Among 2,231 deaths, 1,882 (84.3%) were from Africa [1]. Some authors estimate that the real number of deaths might be as high as 95,000 per year [2]. In the last twenty years, sub-Saharan Africa, and especially the Great Lakes Region, has suffered the highest disease burden [3–6]. Uganda is located in the Great Lakes Region and reports cholera cases and deaths annually since 1998 [7–11]. Communities most vulnerable to cholera in Uganda are situated along the lakes [8,9], where the major economic activity is fishing or fish selling. Fish export rank third as a contributor to the Ugandan Gross Domestic Product (GDP) [12]. Fishermen in Uganda like those in other parts of Africa are poor with high rates of infectious diseases, such as human immunodeficiency virus (HIV) infection [13–15]. While the proximity to water bodies is a known risk factor [4,6,16], little is known about people inhabiting these at-risk areas and so far no interventions targeting fishing villages have been proposed. Generally, WHO recommends integrated cholera prevention and control interventions comprising Water, Sanitation and Hygiene (WASH) measures and Oral Cholera Vaccine (OCV) use in endemic settings. For this article we collected and reviewed epidemiological data on cholera with a focus on fishing villages for the period 2011 to 2015. Our aim was to generate information on the burden and characteristics of cholera epidemics so as to make recommendations on how to target cholera control interventions as recommended by WHO. Given the importance of artisanal fishing throughout sub-Saharan Africa, we expect that our findings will be relevant to other fishing villages in Uganda, the Great Lakes Region and similar settings across Africa.

## Materials and methods

### Ethics statement

The data used in this study were collected as part of routine MOH disease surveillance work. Permission to conduct the study was obtained from the Makerere University School of Public Health Institutional Review Board (IRB 00011353). Surveillance forms were anonymized to ensure confidentiality.

### Study design

We collected, reviewed and documented epidemiological and socioeconomic information on cholera outbreaks in the fishing villages known for frequent cholera outbreaks in Uganda for the period 2011–15 focusing on the outbreaks along African Great Lakes in Uganda and river Nile.

Additionally, we performed household surveys in 2015 to get better understanding of the socioeconomic characteristics and practices of cholera affected households in urban and rural fishing communities.

### Data collection

We worked through Ugandan Ministry of Health (MOH) which routinely conducts disease surveillance and response to outbreaks of cholera and others diseases/events nationwide [17]. Based on this surveillance, the health workers at community, health facility, sub-district, district and central MOH levels investigate potential public health threats or outbreaks and take appropriate preventive and control actions.

The investigation teams and district level units report to central level in Kampala on actions taken and gaps that need additional support. Delay in reporting and response are determined by the public-health relevance of the reported event.

Since 2000, the MOH requires districts to regularly compile weekly reports from their health facilities and aggregate the data into a district summary report that is submitted to the central level in Kampala and used to produce a national level weekly surveillance report [18]. In 2015, Uganda had 112 districts which regularly compiled weekly reports: http://health.go.ug/content/weekly-epidemiological-bulletins. In regards to cholera, more detailed individual case information (line list) is collected from the patient registers and sent to the district and central (national) level MOH head office in Kampala.

Information on the line lists includes: patient name, age, sex, place of origin (district, sub-county (SC), parish, village–from larger to smaller geographic unit), symptoms and signs, date of illness onset, date of admission, treatment administered, date of discharge and outcome.

**Fig 1:**
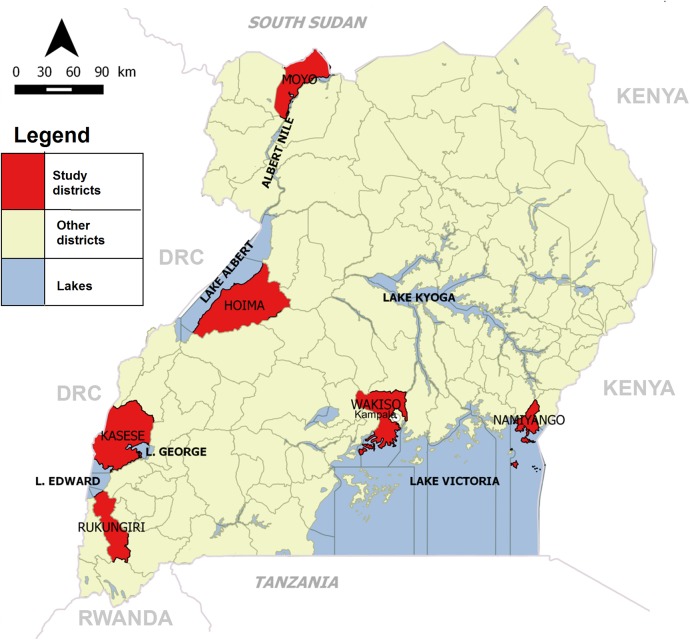
Location of the study districts, major lakes and rivers in Uganda, 2011–2015.

For this study, we reviewed weekly nationwide cholera surveillance reports, laboratory and outbreak investigation reports, and available line lists from the MOH Uganda for 2011–2015. We also assessed for seasonal pattern of the cholera outbreaks using satellite rainfall data (precipitation) for Uganda that was obtained from Weatherbase at http://www.weatherbase.com/weather [19]. We used the long-term average rainfall (precipitation) collected for 101 years from 80 cities/districts in Uganda. We focused on cholera outbreaks that occurred in the fishing villages along the Great Lakes (lakes Victoria, Albert, Edward, George and Kyoga) and along the River Nile (Fig 1).

In addition to the routine MOH disease surveillance system, Uganda with other eight (8) sub-Saharan African countries participated in the African Cholera Surveillance Network (*AFRICHOL*) project which carried out cholera case-based surveillance in selected surveillance areas [20]. In Uganda, these areas included Kasese district in Western Uganda plus Mbale, Busia, Manafwa, Butaleja and Tororo districts in Eastern Uganda.

The patients in Hoima and Kasese districts were followed up within two weeks of discharge from the Cholera Treatment Centre (CTC) by a joint MOH and Makerere University School of Public Health team to collect detailed information on the household characteristics, home environment and implementation of cholera preventive measures. One urban fishing community (Katwe Kabatoro Town Council (KKTC) in Kasese district) and one rural fishing community (in Albertine rural Hoima, Hoima district) were followed up. The information was collected using a structured questionnaire administered to the heads of the households by trained health workers mainly the health assistants from the nearest health facilities.

During home visits, health education and referral of new identified cholera cases was carried out. In addition, information on the characteristics of cholera affected household to understand sanitation and hygiene practices, availability of safe water, socioeconomic status, education level, knowledge and behaviors was collected.

#### Case definitions

We defined cholera cases based on the Ugandan national guidelines (2007), which were adapted from WHO guidelines for cholera prevention and control [21] as follows:

- Suspected cholera: in an area where the disease is not known to be present, a patient aged 5 years or more who develops severe dehydration or dies from acute watery diarrhea or in an area where there is a cholera epidemic (based on laboratory isolation of *Vibrio cholerae* organism and official outbreak declaration), the occurrence in a patient aged 2 years or more of acute watery diarrhea, with or without vomiting.
- Confirmed cholera: isolation of *Vibrio cholerae* O1 or O139 from a stool sample or rectal swab of any patient / dead body with acute watery diarrhea.
- Cholera outbreak: one or more confirmed cholera cases.

### Inclusion criteria

We included only confirmed cholera outbreaks reported to MOH and occurring in the fishing villages along the Great lakes (lakes Victoria, Albert, Edward, Kyoga, George and Katwe) or River Nile with available line lists during the study period 2011–15. Additional inclusion criteria were:

Availability of weekly district surveillance reports, outbreak investigation reports during the study period and laboratory reports confirming the outbreak (at least one confirmed case) through the isolation of *Vibrio cholerae* from stool samples.

### Data for comparison with other communities in Uganda

We collected and reviewed information on the total aggregated number of reported suspected cholera cases and deaths at national level during the same time period from all districts in Uganda by a analysis of the weekly epidemiological reports available at MOH, Kampala.

### Laboratory procedures

Stool collection and testing were conducted as recommended by the MOH laboratory and cholera prevention guidelines [21,22]. Stool samples for laboratory testing were collected from patients meeting the standard case definition for cholera before antibiotic administration. Only 10–20 stool samples were collected in accordance with national (Uganda) laboratory guidelines to confirm the cholera outbreak. A few additional random stool samples were later collected to monitor antimicrobial sensitivity. Finally, the collection of additional stool samples was done to determine the end of the outbreaks.

To transport cholera stool samples, fresh stool samples or cotton tipped rectal swabs soaked in fresh liquid stools were individually placed in Cary-Blair transport media, and then sealed in sterile zip lock bags. The bags were packed into cool boxes, and transported to the Uganda National Health Laboratory Services (Central Public Health Laboratories, http://www.health.go.ug/content/central-public-health-laboratorycphl) in Kampala within seven days for cholera culture identification using standard laboratory cholera identification procedures [23].

### Data management, analysis and interpretation

Data were collected by district health workers after training on study protocol and tools. Data with personal identifiers were coded or made anonymous. The data were entered, cleaned, stored and analyzed using Stata 12 software (StataCorp, Texas, USA) and Microsoft Excel spreadsheets. The differences between groups were tested using chi-square test for proportions (or Fisher exact test for small size). Comparison of age between outbreaks was tested for significance with Kruskall-Wallis test. The analysis of factors associated with cholera deaths was performed using logistic regression models. Variables with p values<0.20 in the univariate analysis were entered into a multivariate model. Variables were kept in the final model if p < 0.05.

For each outbreak, attack rates (number of cases/population) were calculated and expressed per thousand, using sub-county population data as the denominator. Sub-county data used was obtained from the Uganda Bureau of Statistics [UBOS], http://www.ubos.org. Population data from the most recent census (2014) [24] was used for outbreaks occurring in 2014 or 2015 (adding district growth rates for 2015 data, based on 2014 census: 2.45%, 4.27% and 6.61% for Kasese, Hoima and Wakiso districts, respectively). For outbreaks that occurred in 2011–2013, population projections derived from UBOS census data of 2002 were used [25].

Information on residence was self-reported by the patients to the health workers at the health facilities. Based on this information, we used Quantum Geographic Information System (QGIS) [26] to create maps. The administrative boundary GIS layer was obtained from UBOS on the Humanitarian Data Exchange website [27] in ESRI shapefile format. The unit of geographic analysis was the sub-county (administrative level 5), which was recorded for each suspected cholera case. This was the minimum mapping unit that corresponded to the population census data conducted by UBOS [24]. Individual sub-counties (SC) on the map were shaded according to the number of cases occurring within them. We applied several amendments to the SC names. In Namayingo district, Buhemba was replaced by Buyinja because, in the GIS layer we used, Buhemba was a parish of the Buyinja SC. Similarly in Wakiso district, Bussi was replaced by Kasanje. For the Hoima 2012 cholera outbreak, one case from Masindi SC (which belonged to Masindi district) did not appear on the Hoima map. There were two cholera cases during the first cholera outbreak in Moyo district in 2014 from Ciforo SC (Adjumani district) who did not appear on the Moyo map.

## Results

### Epidemiological profiles of the outbreaks

**Fig 2:**
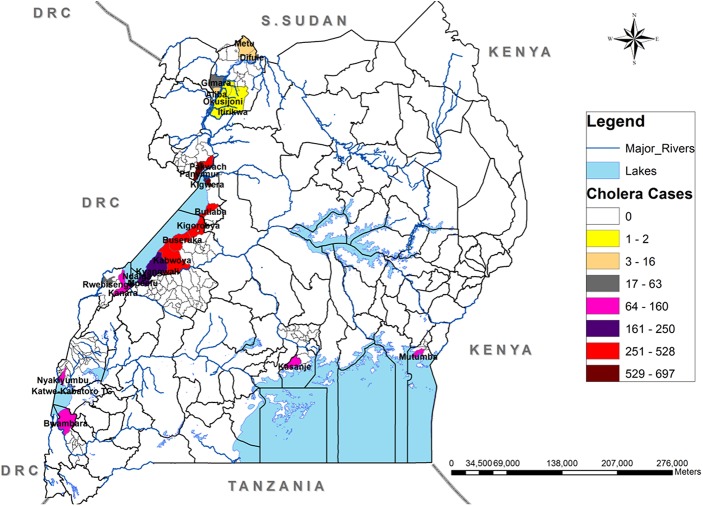
Location of the fishing villages in Uganda that reported cholera outbreaks, 2011–2015.

During the study period 2011–15, a total of 5,059 suspected cholera cases and 113 deaths (CFR: 2.2%) were recorded. There were several outbreaks recorded in 12 districts of Uganda with fishing villages as in Fig 2.

In six out of 12 districts that reported cholera outbreaks there were ten (10) outbreaks for which detailed information was collected for the period 2011–2015. All reported cholera outbreaks in fishing villages during the study period were located along the African Great Lakes (Victoria, Albert, Edward and Katwe) or the River Nile. There were no cholera outbreaks in the fishing villages on Lakes Kyoga and George.

**Fig 3:**
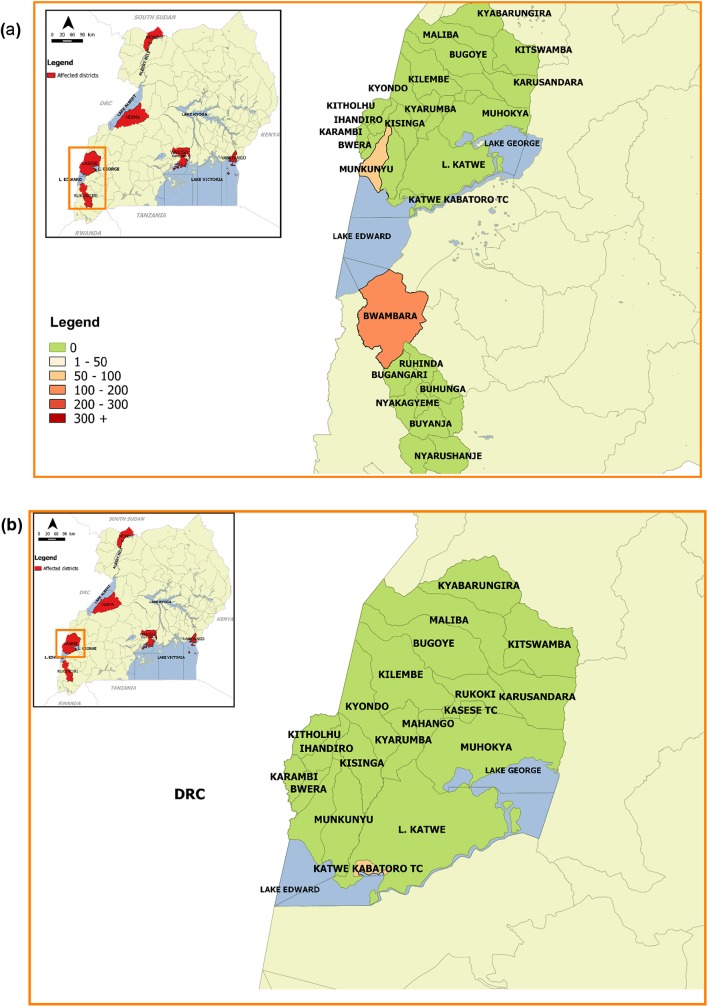
Cholera outbreaks that occurred in fishing villages along Lake Edward. Fig 3a: Cholera outbreaks in Rukungiri and Kasese districts, 2011; Fig 3b: Cholera outbreak in Kasese district, 2015.

Of the ten (10) cholera outbreaks with detailed information, three (3) were reported in fishing villages on Lake Edward: one in Rukungiri district in 2011 (Bwambara SC, Rwenshama parish) and two in Kasese district (Nyakiyumbu SC, Kayanzi parish in 2011 and Katwe-Kabatoro Town Council [KKTC] SC, Kyarukara parish in 2015) (Fig 3).

**Fig 4:**
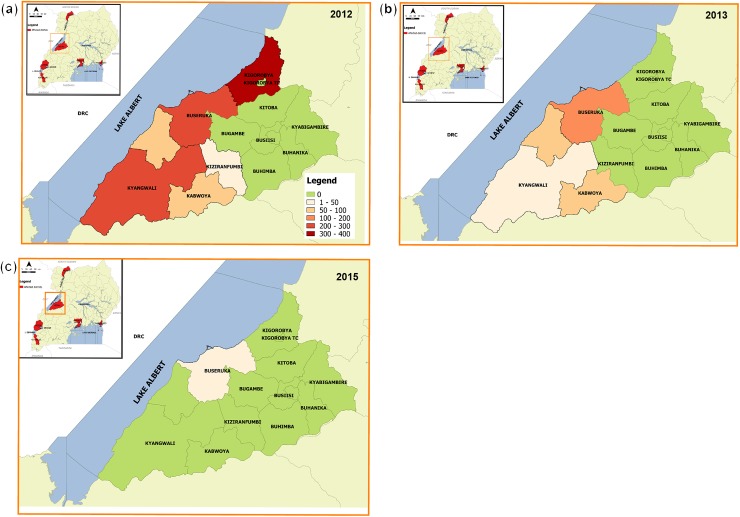
Cholera outbreaks that occurred in fishing villages along Lake Albert. Fig 4a-4c: Cholera outbreaks in Hoima district in 2012, 2013 and 2015, respectively.

Three (3) cholera outbreaks were also reported on Lake Albert in the district of Hoima in 2012, 2013 and 2015 (Fig 4).

**Fig 5:**
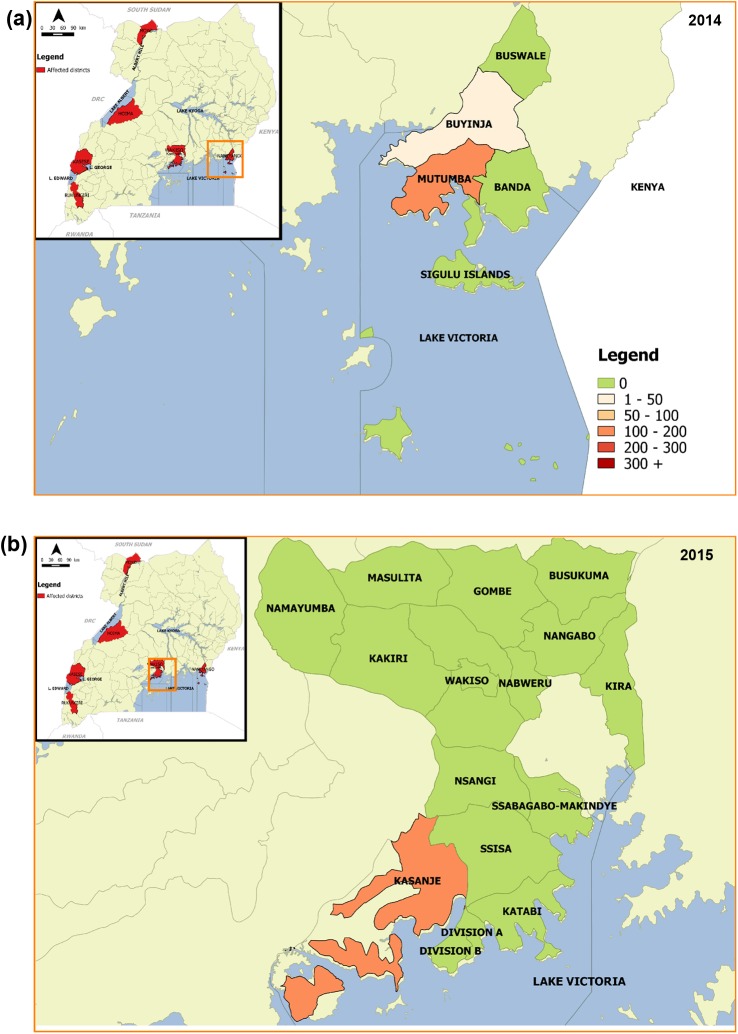
Cholera outbreaks that occurred along Lake Victoria. Fig 5a: Cholera outbreak in Namayingo district, 2014; Fig 5b: Cholera outbreak in Wakiso district, 2015.

Along Lake Victoria, two (2) cholera outbreaks were reported in the districts of Namayingo in 2014 (mainly Mutumba SC, Lubango parish) and Wakiso in 2015 (on the islands of Bussi / Kasanje SC) (Fig 5).

**Fig 6:**
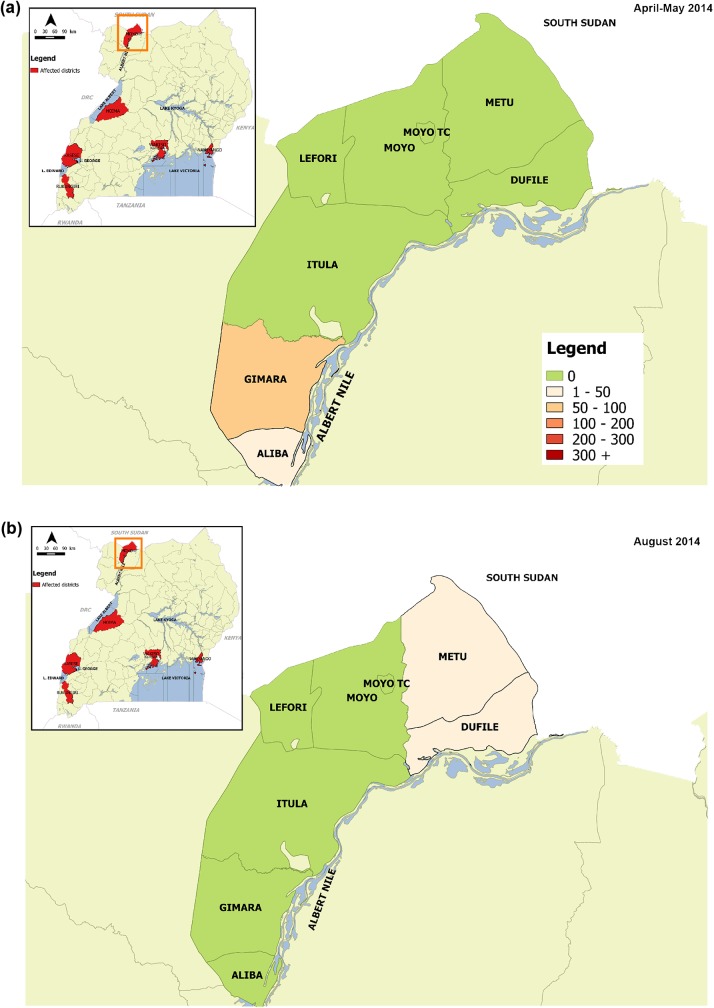
Cholera outbreaks that occurred in fishing villages along the Nile River. Fig 6a: Cholera outbreak in Moyo district, April-May 2014; Fig 6b: Cholera outbreak in Moyo district, August 2014.

In addition to the three affected lakes, cholera outbreaks were reported along the River Nile (Albert/White Nile) in 2014 in the fishing communities in Moyo district (Fig 6).

Over the study period, the total number of reported cholera cases and deaths from these outbreaks on the line lists were 1,827 and 43 respectively (Case-Fatality Ratio [CFR], 2.4%). There were a limited number of affected sub-counties in each outbreak (between 1–5 sub-counties), representing 0.8% to 47.4% of the total district population. Half of the outbreaks occurred in only one SC within the affected district. Some sub-counties in Hoima district along Lake Albert had recurrent outbreaks, in particular Buseruka, which was affected by cholera in 2012, 2013 and 2015. Within Buseruka SC, Tonya was the most affected parish contributing 96.3% of all cholera cases in the SC.

**Fig 7:**
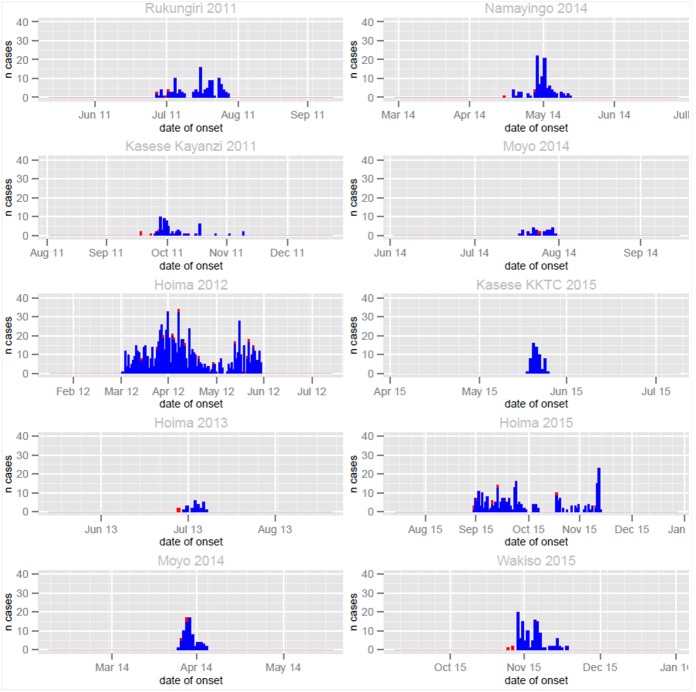
Cholera cases and deaths by year of the outbreak in the fishing villages, 2011–2015. Legend: blue color, suspect cholera cases; red color, cholera deaths.

Overall, cholera outbreaks lasted an average of 35 days (5 weeks) with a range of 8–90 days. Fewer cholera cases occurred during January-March, while April-May had the highest proportion of total cases (22.5%). The majority of the outbreaks occurred during the rainy season (82.4%) with a big peak during the months of April-June and a smaller peak during September–November of each year (Fig 7).

Attack rates in affected sub-counties ranged from 0.2 to 9.1 per 1000 (Table 1). The highest attack rate was in Katwe-Kabatoro Town Council (KKTC), where no deaths were reported.

**Table 1:** Description of cholera outbreaks in fishing villages in Uganda, 2011–2015.

Out-break	Year	District	District pop.	Num. cases	Num. deaths (CFR)	Most affected sub-counties (SC)	Num. of cases in the most affected SC (%total)	SC pop. (% of district population)	Attack rate at SC level (per 1,000)	Outbreak duration (days)	Total pop. affected by outbreak / district pop (%)
1	2011	Rukungiri	316,400	123	3 (2.4%)	Bwambara	123 (100%)	29300 (9.3%)	4.2	32	9.3%
2	2011	Kasese	721,400	69	4 (5.8%)	Nyakiyumbu	69 (100%)	31340 (4.3%)	2.2	53	4.3%
3	2012	Hoima	548,800	909	17 (1.9%)	Kigorobya	331 (36.4%)	65410 (11.9%)	5.1	90	47.4%
						Buseruka	266 (29.3%)	41136 (7.5%)	6.5		
						Kyangwali	224 (24.6%)	93107 (17.0%)	2.4		
						Kabwoya	82 (9.0%)	60357 (11.0%)	1.4		
4	2013	Hoima	575,100	26 (60*)	2 (3.3%*)	Buseruka	26 (100%)	43108 (7.5%)	0.6 (1.4)*	11	7.5%
5	2014	Moyo	137,489	74	3 (4.1%)	Gimara	61 (82.4%)	13294 (9.7%)	4.6	11	21.7%
						Aliba	11 (14.9%)	16582 (12.1%)	0.7		
6	2014	Namayingo	223,229	109	2 (1.8%)	Mutumba	102 (93.6%)	42311 (19.0%)	2.4	29	33.1%
						Buhemba	7 (6.4%)	31472 (14.1%)	0.2		
7	2014	Moyo	137,489	29	1 (3.5%)	Dufile	16 (55.2%)	8983 (6.5%)	1.8	14	27.1%
						Metu	13 (44.8%)	28236 (20.5%)	0.5		
8	2015	Kasese	719,229	60	0 (0%)	KKTC	60 (100%)	6568 (0.9%)	9.1	8	0.9%
9	2015	Hoima	598,409	282		Buseruka	194 (68.8%)	44855 (7.5%)	4.4	76	35.5%
					8 (2.8%)	Kabwoya	59 (20.9%)	65813 (11.0%)	0.9		
						Kyangwali	28 (9.9%)	101524 (17.0%)	0.3		
10	2015	Wakiso	2,068,533	146	6 (4.1%)	Bussi	145 (99.3%)	17548 (0.8%)	8.6	25	0.8%

CFR: case-fatality ratio; SC: sub-county; KKTC: Katwe-Kabatoro Town Council. *Incomplete line list: total number of cases = 60 and deaths = 2 according to the outbreak report. With n = 26, the attack rate is 0.6 ‰, whereas using the total number of cases (n = 60), the attack rate is 1.4 ‰. CFR was calculated using the total number of cases and deaths (2/60).

**Fig 8:**
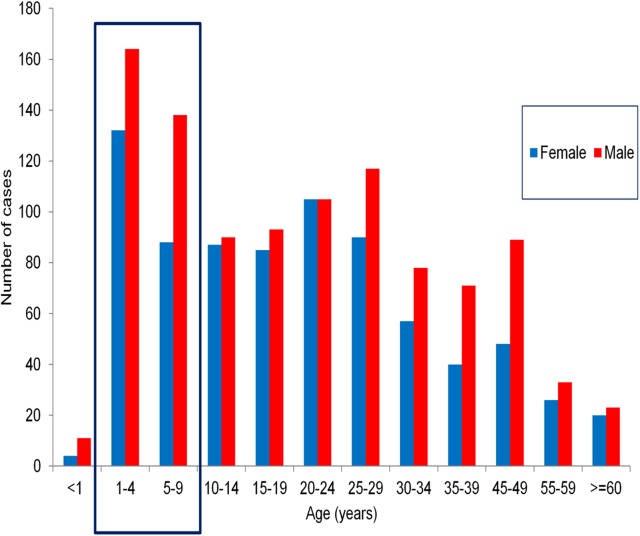
Reported cholera cases by age group and gender in fishing villages in Uganda, 2011–2015.

Male to female sex-ratio was 1.3 but varied greatly between outbreaks (from 0.7 in Moyo to 2.0 in Kasese/Kayanzi village). Overall, males were more affected than females in all age groups (Fig 8).

The mean age was 21.2 years (range 0.1–81) and median age 20 years (Interquartile range [(IQR] 7–30), with significant differences between outbreaks (p<0.001) from a mean age of 18 years in Namayingo district in 2014, to mean age of 40.2 years in Moyo district during the second outbreak in 2014. A total of 537/1,827 cases (29.4%) were children below age 10 years, with 311 children under 5 years and 226 children age 5–9 years. Outbreaks with the highest proportions of under the age of 10 years were in Hoima district during 2015 (37.2%), 2013 (34.6%) and 2012 (34.1%) and the 2015 Kasese KKTC outbreak (36.7%).

Nine (9) of ten outbreaks had a CFR>1% (Table 1). The CFR was significantly higher among males than females (3.2% vs. 1.3%, p = 0.02) and in six out of ten outbreaks, no deaths among females were notified. The CFR also varied between outbreaks within a district, e.g. in Kasese from 0% in the urban area of KKTC in 2015 to 5.8% in Kayanzi fishing village during 2011. There was a higher average CFR (= 3.4%) among adults aged >25 years based on available line lists.

Forty-two percent (40%) of deaths occurred within the first week of the outbreaks. Factors independently associated with death in the multivariate analysis were gender (male vs. female OR = 2.6 (CI 95%, 1.2–5.6)) and month of onset (reference April, OR July = 19.2 (CI 95%, 4.9–75.6), OR September = 12.3 (CI 95%, 2.2–69.6)).

Laboratory tests showed that *Vibrio cholerae* O1 was responsible for all outbreaks. The two serotypes *Inaba* (8 out of 10 outbreaks) and *Ogawa* (responsible for 2 out of 10 outbreaks, in Namayingo and Wakiso districts) were isolated from stool samples. Regardless of serotype, the strains were sensitive to *cefuroxime*, *tetracycline*, *erythromycin and ciprofloxacin* and resistant to *nalidixic acid*, *cotrimoxazole and chloramphenicol*.

### Environmental conditions and characteristics of households with cholera cases

Outbreak investigation reports indicated that epidemics in different locations within fishing villages and periods presented similarities regarding environmental, personal and household characteristics.

Common findings in the outbreak investigation reports were the low latrine coverage of less than 50%, open defecation and bathing in lake waters by the communities in the fishing villages.

A total of 137 households (51 in KKTC-Kasese district, and 86 in Hoima district) were visited within two weeks of discharge of cholera cases by the health assistants to collect socio-economic and environmental information on the households.

Majority of household heads in Hoima district were illiterate (61%, 50/82) with the average monthly household income of USD 37, and 60% had less than USD 30 per month. The most common source of drink water was lake Albert (72%, 62/86). The majority (64%) of households stored water in open containers and 18% practiced open defecation. Less than half (42.3%) knew how to treat cholera using Oral Rehydration Salt (ORS).

In contrast, KKTC (urban area), the majority of households got their drinking water from the protected springs and stored it in covered containers (100%), had community latrines for the homesteads (98%) and knowledge on cholera treatment with ORS was 84%.

### Comparison of cholera cases in fishing villages with other communities in Uganda

Nationwide reported cholera data during the study period showed that outbreaks in fishing villages were responsible for an average of 58% of cholera cases and 55% of deaths in Uganda (Table 2).

**Table 2:** Annual reported cholera cases and deaths in Uganda by district and by affected community, 2011–2015.

Year	Annual cases	Annual cases in fishing comm	Annual national deaths (CFR, %)	Fishing comm deaths (CRF, %)	Districts with deaths in fishing community	% annual cholera cases in fishing community	% annual deaths in fishing community
2011	229	192	7 (3.1)	7 (3.6)	Rukungiri and Kasese	84%	100%
2012	6,226	3,579	135 (2.2)	68 (1.9)	Hoima, Bulisa,Kibaale, Nebbi and Ntoroko	57%	50%
2013	751	535	26 (3.5)	19 (3.6)	Nebbi, Bulisa, Hoima,	71%	73%
2014	322	262	9 (2.8)	7 (2.7)	Moyo, Namayingo and Nebbi	81%	78%
2015	1,270	491	29 (2.3)	12 (2.4)	Wakiso and Hoima	39%	41%
**Total**	**8,798**	**5,059**	**206 (2.3)**	**113 (2.2)**	** **	58%	55%

Majority of the ten outbreaks in Fig 7 above occurred in the rainy season. This seasonal pattern is much clearer for the aggregated overall national annual cholera cases that occurred during the study period 2011–15 in fishing villages in Uganda.

**Fig 9:**
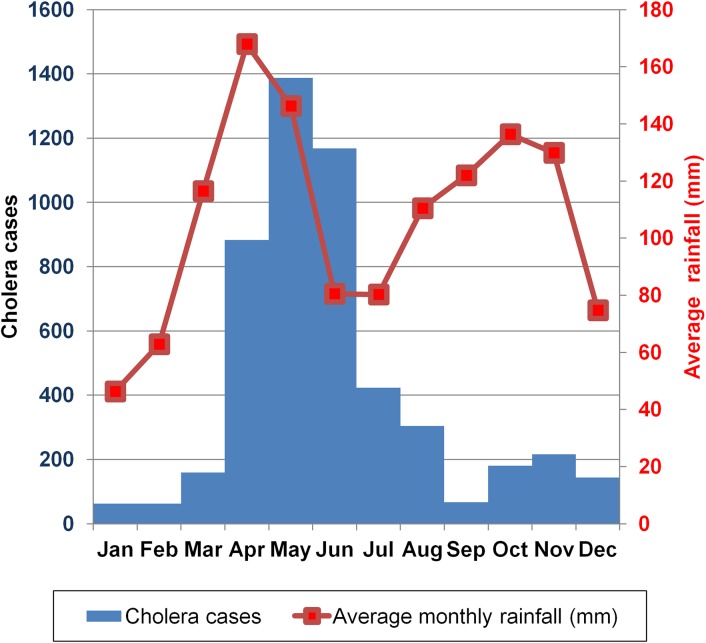
Monthly reported cholera cases in fishing villages and average long term rainfall in Uganda, 2011–2015.

There were two peaks that corresponded to bimodal rainfall seasonal pattern. The highest cholera peak stretches from April-August and smaller one in October–December. Cholera peaks lagged behind the rainfall peak with an average of 5–6 weeks. The average monthly rainfall and the number of reported cholera cases during the study period in fishing villages are shown in figure (Fig 9).

### Control measures and challenges experienced by responders

The MOH used a multi-sectoral approach to implement all or some of the following interventions: disease surveillance; patient care; infection control; health education; promotion of latrine construction and use; promotion of hand washing; enforcement of sanitation and hygiene using strategies such as sanitation improvement campaigns; decongesting the lake by reducing the number of illegal boats; and imposing a ban on the sale of cold foods.

The most common challenges were: lack of alternative water source (other than the lake); inadequate hand washing facilities; ignorance regarding the importance of hand washing; difficulty in implementing sanitation measures since many of the community members had a livelihood based on the mobile profession of fishing; and preference for open defecation due to the perception that it increased fish catch. Some homes were located close to the lakeshore making it difficult to construct latrines near to residences.

The high level of poverty impeded effective cholera control interventions. For example, in Kasese district repair/maintenance of safe water systems could not be done due to failure to mobilize adequate money from the community.

Also, many patients reported late for medical care leading to poor outcome. Finally, language barriers between actors and cholera affected populations since the languages spoken by the affected communities were not the languages used on the local radio messages to mobilize the communities.

## Discussion

Our study showed that cholera is a serious public health problem contributing to morbidity and mortality among the fishing communities in the Great Lakes Region of Africa and Uganda specifically. We systematically documented and characterized cholera outbreaks in these important yet underserved communities, which constitute less than 4.3% of the total Uganda population [28] but reported over 50% of the annual cholera cases and deaths.

A key motivation for the study was to identify populations for targeted interventions. In this respect, among the 1,827 cases documented, children under age five years and 5–9 years (school going age) were the most affected age groups. Moreover, in some communities such as Hoima district, cholera was endemic with well-defined locations or “hotspots” that were repeatedly affected in the sub-counties of Buseruka, Kyangwali, and Kabwoya.

This study is in agreement with earlier findings of cholera endemicity in some of these communities such as in Hoima district where cholera occurred almost annually [8]. Conversely, no cholera outbreaks were reported on Lake Kyoga, the second largest lake, and one with a large fishing community. The protective factors in Lake Kyoga fishing villages need to be studied. Furthermore, outbreaks in fishing communities may be the starting point for epidemics in neighboring areas, including in communities with high population density, poor sanitation, and inadequate access to safe water and hygiene.

Most cholera cases occurred during the rainy season (82%), with cholera peaks occurring 5–6 weeks after the rainfall peak, possibly because time was needed for the water sources to get contaminated by rains before initiating the outbreaks.

The CFRs were higher than those recommended by WHO but similar to those documented at country-wide levels (2.4% vs 1.6–2.2%) [11] and to what was seen through the *AFRICHOL* enhanced surveillance in specific zones of Uganda (2.5%, mainly Kasese and Mbale districts) [20]. The CFR was 2.5-fold higher among males than females possibly because males fell ill while on the lakes fishing and in places distant from treatment facilities.

The communities in the fishing villages, in addition to living in poor sanitation conditions, lack safe water. Artisanal fishing obliges fishermen to be mobile and to frequently have different residences throughout the year. These temporary settlements are aggravating factors for cholera spread due to difficulties for the individuals to control their environment and access sanitation facilities and clean water. The repeated cholera outbreaks in these communities may also be due to the high level of poverty, inadequate access to social services and high illiteracy levels, as seen in other African countries [13,29]. The average household income in Hoima district among households reporting cholera cases was USD 37 per month, which was three times lower than the average nominal income in Uganda’s mid-West sub-region of USD 110 during the study period [30] but similar to incomes among other informal workers in Uganda [31]. Also, like elsewhere in the sub-Saharan African sub-region, many lakes are shared between countries, and communities living on them have inadequate provision of social services. In Uganda this scenario was worsened by sharing the western international border with a cholera endemic zone in DRC [32] and a challenge of cross-border cholera outbreaks in the region [33].

Studies indicate that these lakes could serve as *Vibrio cholerae* reservoir from where cholera later spreads to other parts of the Great Lakes Region [4,34–37].These factors put the fishing communities at high risk of cholera transmission in addition to their known vulnerability for other infectious diseases such as HIV [38,39].

The government of Uganda has implemented Universal Primary Education (UPE) since 1996 as main policy tool for poverty reduction and human development [40]. However, our findings indicate that communities in the fishing villages have not been able to utilize this opportunity. Consequently, future efforts should assess how to achieve better integration of existing programs as well as development of tailored programs for fishing villages and other cholera vulnerable communities.

### Study limitations

These findings reflect available data from the MOH regarding cholera outbreaks occurring in often remote lakeshore communities. They may not be exhaustive, so the proportion of those cases among country-wide cases occurring in the same period is possibly underestimated. Few variables were available in the line lists, which did not allow us to perform detailed analysis of risk factors at individual level. Because we could not include control conditions, we are unable to comment on the degree to which specific factors contributed to the occurrence of cholera outbreaks in the fishing villages under the study. Therefore a follow up a case-control or cohort study should be carried out to better inform the global community on this issue.

Finally, few laboratory reports were available making it difficult to draw firm conclusions of cholera serotypes or antibiotic resistance. Furthermore, due to inadequate laboratory facilities and techniques in most of the affected communities, isolation and storage of the cholera organism for advanced testing such as molecular characterization could not be done. In order to conduct such tests in future, Uganda government and local partners should institute a feasible mechanism to safely transport stool samples and or store them securely.

## Conclusions

Cholera is a big public health problem in the fishing villages along the Great Lakes and River Nile in Uganda. In the short term, a comprehensive cholera prevention and control approach as recommended by WHO [41,42] that includes complementary use of OCV to Water, Sanitation and Hygiene (WASH) interventions (latrine construction and use, water chlorination, hand washing campaigns with soap, compact water filtering pumps) and others should be instituted. This approach could provide economic benefits by boosting tourism and food exports which shall reduce the poverty levels and the cholera disease burden in the fishing villages and Uganda.

Because cholera is seasonal, the periods without outbreaks provide an opportunity to intervene and prevent future outbreaks. This preventive approach is particularly appealing since the short duration of most outbreaks makes a reactive approach very challenging. Targeting smaller geographic areas, and high-risk groups within these areas, may provide an efficient means of reducing overall cholera burden. Further studies namely; a case control study, studies to assess community knowledge and practices regarding cholera prevention and molecular typing should be conducted. In the long term, improving income, education, and living conditions in the fishing villages will provide the best means of reducing cholera and other diseases of poverty.
